# Simplified Method to Predict Mutual Interactions of Human Transcription Factors Based on Their Primary Structure

**DOI:** 10.1371/journal.pone.0021887

**Published:** 2011-07-05

**Authors:** Sebastian Schmeier, Boris Jankovic, Vladimir B. Bajic

**Affiliations:** Computational Bioscience Research Center (CBRC), King Abdullah University of Science and Technology (KAUST), Thuwal, Kingdom of Saudi Arabia; Centro de Investigación Príncipe Felipe, Spain

## Abstract

**Background:**

Physical interactions between transcription factors (TFs) are necessary for forming regulatory protein complexes and thus play a crucial role in gene regulation. Currently, knowledge about the mechanisms of these TF interactions is incomplete and the number of known TF interactions is limited. Computational prediction of such interactions can help identify potential new TF interactions as well as contribute to better understanding the complex machinery involved in gene regulation.

**Methodology:**

We propose here such a method for the prediction of TF interactions. The method uses only the primary sequence information of the interacting TFs, resulting in a much greater simplicity of the prediction algorithm. Through an advanced feature selection process, we determined a subset of 97 model features that constitute the optimized model in the subset we considered. The model, based on quadratic discriminant analysis, achieves a prediction accuracy of 85.39% on a blind set of interactions. This result is achieved despite the selection for the negative data set of only those TF from the same type of proteins, i.e. TFs that function in the same cellular compartment (nucleus) and in the same type of molecular process (transcription initiation). Such selection poses significant challenges for developing models with high specificity, but at the same time better reflects real-world problems.

**Conclusions:**

The performance of our predictor compares well to those of much more complex approaches for predicting TF and general protein-protein interactions, particularly when taking the reduced complexity of model utilisation into account.

## Introduction

The transcriptional regulatory machinery that acts on the transcription of genes is complex and not yet completely understood. Transcription factors (TFs) are proteins that regulate transcription initiation of genes by binding to regulatory regions on genomic DNA [1]. They exert their function in the nucleus of cells where they often work cooperatively through the formation of TF complexes to enhance or repress transcription initiation [2], [3]. To better understand the elaborate transcriptional machinery that acts within the cell nucleus, it is essential to determine these TF interactions. The combinatorial regulation of transcription initiation has been studied extensively [4]–[9], where groups of TFs that work cooperatively in the transcription of genes or gene groups were identified. The combinatorial regulation described in [4]–[9] does not necessarily entail the physical interaction of the participating TFs although this is frequently required.

Protein-protein interaction (PPI) prediction has gained much attention over the last decade. Various methods and tools for the prediction of pairs of proteins that can interact have been developed [10]–[14]. These methods make use of manifold properties of proteins and combinations thereof, such as functional categorisation and gene ontology annotations [15], primary structure [16]–[20], secondary, tertiary structure, and protein domain information [11], [12], [14], [21]–[24], ortholog-based and phylogeny-based profiles [25], [26], gene expression and other experimental data [27], as well as text mining [13], [28].

Predicting TF interactions can be seen as a subclass of the general PPI prediction problem that is more complex, because members of TF families are often sequence-wise similar to each other [29]. Deriving prediction models from data sets with similar examples is inherently difficult [30], [31]. Furthermore, TFs exert their function as regulatory proteins of transcription initiation in the same cell compartment, the nucleus, making it impossible to utilise cellular localisation as a criterion in prediction. Many of the published PPI prediction methods use exactly this localisation feature to construct negative TF pairs. If models derived in such manner have higher accuracy, this may be attributed to potentially disparate characteristics of proteins from different cellular locations (e.g. mitochondrial proteins and those functional in the cell nucleus) thus making classification easier. Examples in our negative set are from the same cellular compartment. Finally, information about known TF interactions is relatively scarce compared to PPIs.

Previous approaches for deciphering the combinatorial control of TFs have included co-expression analysis [32], thermodynamic models based on time-course microarray data [33], relationships of TF binding sites (TFBSs) [34], [35], and combinations of these methods [36]. To aid future research on combinatorial gene regulation, our study presented here aims at predicting TF interactions computationally. As with computational PPI predictions, a good representation (e.g. feature vector) for an interacting TF pair has to be found. The former mentioned methods used information for such representations that is often difficult to acquire. To circumvent this obstacle, the approach we applied here is based on protein sequence information alone, which significantly simplifies application of our method. The present analysis shows that even with limited prior knowledge about TFs and under stringent conditions imposed on the system, it is possible to achieve prediction performance that compares well to those of more complicated approaches. Our analysis utilises amino acid (AA) properties of TF primary sequences and combines these into a representation for TF pairs. The artificial intelligence system employed to predict if a pair of TFs can interact is based on quadratic discriminant analysis (QDA) [37]. We employed a 10-fold cross-validation (CV) to select the most discriminative set of features used for building the predictive model in order to reduce model complexity and improve its robustness. We used forward feature selection method with a wrapper [38]. This process identified 97 such model features out of more than 3000. The 10-fold CV shows that the expected accuracy of the system we propose here achieves 82.04%. An evaluation of the final model on a completely blind set (i.e. a set not used in training and CV) revealed an accuracy of 85.39% (specificity of 83.93% and a sensitivity of 86.92%).

## Methods

### Data Preparation

The set of human TFs from a current collection, based on DNA-binding protein domains, was extracted [39]. In addition, 70 additional proteins from TRANSFAC Professional database (version 11.4) [40], [41], which were not in the aforementioned list of TFs, were hand-curated. After manual inspection, 34 of these were included in the list of TFs. Finally, all identifiers were mapped to Uniprot identifiers. The final set of TFs consisted of 1,372 TFs.

PPI were extracted from four public interaction databases (MINT [42], IntAct [43], BioGRID [44], and Reactome [45]). Only interactions of the following PSI-MI (Molecular interaction standard of the Proteomics Standards Initiative) types were taken into consideration [46]:

- MI:0195 (covalent binding)
- MI:0407 (direct interaction)
- MI:0915 (physical association)

Using TFs from above as a foundation, 1,237 TF interactions were extracted from the four databases. The number of TFs comprising these interactions was 508.

Examples of false (negative) TF interactions were generated by randomly associating two TF entities from the 1,372 TFs. Three different classes of negative TF pairs were generated based on information in the above-mentioned databases:

- ‘Absolute’ negatives: TF-TF pairs are generated from TFs not known to interact with any other protein.
- ‘Partial’ negatives: TF-TF pairs are generated taking one TF that is known to interact with other proteins, while the other TF is taken from the group of those not known to interact with other proteins.
- ‘PPI negatives’: Both TFs that form a TF-TF pair are known to interact with other proteins, but the pair itself is not known to mutually interact.

Three groups of such presumed negative interactions were generated. 412 TF interactions from each group were selected at random, resulting in a set of 1,236 negative TF interactions. These were comprised out of 1,147 different TFs. During training, negative examples were drawn from the three groups described above. Each group contained 412 samples, thus, forming the negative training set of 1236 samples.

A BLAST [47] database of all sequences of the TFs that comprise the positive (known) interactions was created. A BLAST search against TFs in the same database was conducted in order to find those TFs with high sequence similarity (based on score and identity values reported by BLAST). All pairs of TFs that had identity greater than 80 percent were selected and then used in the following way: Let *A* and *A′* represent two TFs with a high sequence similarity. Common binding partners between these in the positive set of interactions were then identified. Let *B* be one such partner for both *A* and *A′* and let them form interactions *A-B* and *A′-B*. One of these was then excluded, for example *A′-B*, from further consideration. In this manner a potential for redundancy of the TFs as participating partners in TF interactions was reduced. Following this reduction strategy, 1,182 interactions in the positive set were retained. The number of TFs comprising these interactions was 508. Applying the same methodology for sequence identity to the negative set of interaction, no cases that warranted exclusion based on sequence identity were found.

### Feature Representation and Feature Vectors

The AAIndex database [48] (URL: www.genome.jp/aaindex/) contains biochemical and physicochemical properties for AAs reported in the scientific literature. In total, the database at the time of download contained 544 AA properties. Only those properties that were available for all 20 AAs were selected for our analysis. This reduced the number of properties to 531 (see Table S1).

The feature vectors were compiled as follows. Consider a sequence *s* of AAs. The representation *F_s_* for *s* consists of 531 features *f_p_*, each representing the mean of one of the 531 AA properties *p* over *s*, *F_s_* = (*f_p1_*,… *f_p531_*). An individual feature *f_p_* for AA property *p* was calculated as the mean value of *p* across AAs in the protein sequence. Thus, any sequence *s*, disregarding its length was represented with the same length of feature representation vector. If an AA in the sequence was “X” or “U”, then the AA was disregarded from the averaging process and the sequence length was correspondingly reduced.

It is known that different areas in a protein sequence serve different purposes, for example, it is known that a protein sequence has a C-terminal and N-terminal part, which for themselves have specific properties [49], [50]. In order to capture these differences to some degree in our model, we split the linear protein sequence into the three parts. After careful consideration it became apparent that a split of 20%, 60% and 20% of the protein sequence yielded reasonable results. The first 20% segment is aimed at representing the functionality of the N-terminus, while the last 20% segment represents in our model the C-terminus. In order to create the feature representation *F_t_* of a single TF *t*, its AA sequence *s* was thus divided into three sections, the start section *ss* (N-terminus), the middle section *sm*, and the end section *se* (C-terminus) comprising of 20%, 60% and 20% of protein length respectively as described. Feature representation was calculated for each section and represented as a vector *F_s_*, with the feature vector of a TF *F_t_* being the concatenated vector of *F_ss_*, *F_sm_*, and *F_se_*:
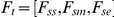
This results in 1,593 features for a single TF. The feature vector for a TF pair *t1*∶*t2* consists of two concatenated feature vectors for each participating TF, *F_t1∶t2_* = [*F_t1_*, *F_t2_*] consisting of a total of 3,186 features (1,593 from each TF comprising the interacting pair). In order to avoid multiple different representations of the same TF interaction pair caused by symmetry of the interaction, the following condition was imposed for concatenation. Consider a TF interaction between *A* and *B*, where *A* and *B* are two TFs, then the first vector is always the one for the TF with the smaller molecular weight. Assume that in the interaction above, *B* is the TF with the smaller molecular weight, then the interaction between *A* and *B* is always expressed as *B*-*A*. In this manner, the resulting feature vector for a TF interaction is always unique.

### Classification algorithm

We used classification based on quadratic discriminant analysis (QDA) [37]. The algorithm is implemented in Matlab® [The MathWorks Inc., Natick, Massachusetts] as a part of its built-in function ‘classify’. Input data were sets of feature vectors for positive and for negative examples.

### Feature Selection and Model Optimization

Our goal was to select the minimal number of features that still provided good classification performance. In order to select features that are most relevant for distinguishing positive and negative examples, forward feature selection [38] by a wrapper algorithm with the QDA-based classifier was used. This was applied in a 10-fold CV that also permitted the estimation of the classifier's performance. In our implementation of the wrapper algorithm the best individual feature was first selected based on the CV performance. Then, to determine the next feature, which would produce the best QDA performance when used in conjunction with the already selected feature, all combinations of the first selected feature with all remaining ones were tested. This testing was performed with the 10-fold CV and the best performing feature was added to the previously selected list. This iterative process was repeated by gradually adding one feature at a time. The feature selected after each iteration step was the one such that the feature list that includes that selected feature averaged best performance across all CV folds compared to other feature candidates. The same data fold partitions were preserved during the process. This allowed for the simultaneous estimation of the QDA classifier performance as well as selection of the best combinations of features. We tested combinations of features up to a total of 150 features.

### Performance Evaluation

We used several performance measures to judge the performance of a classification system [51]. Table 1 shows a confusion-matrix of possible outcomes of a prediction with respect to the actual class of the classified example. The performance measures of the classifier are defined in Table 2.

**Table 1:** Confusion Matrix.

		Actual class	
		Positive	Negative
**Predicted class**	**Positive**	True positive (TP)	False positive (FP)
	**Negative**	False negative (FN)	True negative (TN)

The table indicates the nomenclature for an outcome of a prediction relative to the actual value.

**Table 2:** Performance measures.

Measurement	Equation
Precision	TP/(TP+FP)
Sensitivity (Recall)	TP/(TP+FN)
Specificity	TN/(TN+FP)
False Discovery Rate (FDR)	FP/(FP+TP)
Accuracy	(TP+TN)/(TP+FP+TN+FN)
F-measure	2 * Precision * Sensitivity/(Precision+Sensitivity)

The table shows the performance measures used. TP: True positives; FP: False positives; TN: True negatives; FN: False negatives.

## Results

**Figure 1:**
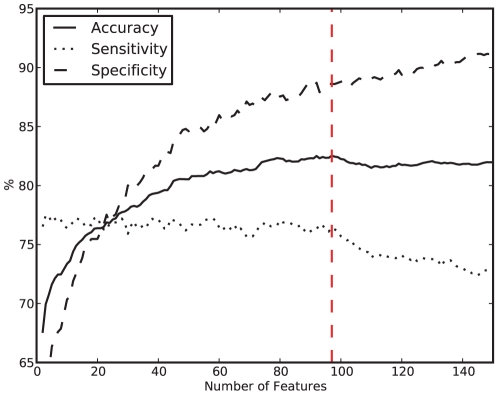
Feature vector length versus accuracy, specificity and sensitivity. The figure shows for different feature vector lengths, selected through the feature selection algorithm explained above, the average accuracy, sensitivity and specificity of the 10-fold CV. The model that uses 97 features (red dashed line) achieves the best accuracy of 82.04% while having a sensitivity of 76.45% and a specificity of 88.61%.

For the complete set of positive (1,182) and negative (1,236) TF interactions (1,182+1,236 = 2,418), all 3,186 features were extracted and the feature vectors created (see Materials and Methods). The set of 2,418 positive and negative TF interaction feature vectors were randomly split into eleven groups, preserving approximately the same ratio of positives and negatives within each group. Ten groups were used for feature selection and model evaluation utilising a 10-fold CV (see Materials and Methods). The eleventh group was retained as a completely independent test set of positive and negative interactions. The feature selection was performed as described in Materials and Methods and the best results were achieved with 97 features (see Figure 1, Table S2). Table 3 shows the results using 10-fold CV with the selected 97 features. The average CV accuracy of the method is 82.04%, while having a specificity of 88.61%, and a sensitivity of 76.45%. Finally, we chose the 97 features that jointly performed best within the CV and we created a model from all feature vectors used for the CV. This model was then applied to the eleventh group of feature vectors that has not been used for either feature selection or creation of the QDA model. On this blind set, our model achieved an accuracy of 85.39% and F-measure of 85.32%, while the specificity was 83.93%, the sensitivity was 86.92%, and the precision was 83.78%.

**Table 3:** Cross-validation results.

Fold	Sensitivity	Specificity	Precision	FDR	Accuracy	F-measure
1	75.21	90.91	91.00	9.00	82.27	82.35
2	80.34	92.16	92.16	7.84	85.84	85.85
3	80.51	88.24	88.79	11.21	84.09	84.44
4	80.34	80.39	82.46	17.54	80.37	81.39
5	75.63	84.31	84.91	15.09	79.64	80.00
6	77.88	90.65	89.80	10.20	84.09	83.41
7	77.12	89.22	89.22	10.78	82.73	82.73
8	68.29	92.78	92.31	7.69	79.09	78.50
9	73.73	91.18	90.63	9.37	81.81	81.31
10	75.42	86.27	86.41	13.59	80.46	80.54
Average	76.45	88.61	88.77	11.23	82.04	82.05

The table shows individual results as well as the average results of the 10-fold CV run using 97 features. FDR: false discovery rate.

## Discussion

The task of predicting TF interactions is comparable to the task of predicting PPIs. Most methods for predicting PPIs use a great deal of information to represent protein pairs for computational inference of their mutual binding. Prediction of general PPIs has been done before only from sequence information to circumvent the obstacles of the requisition of the multitude of other data [16]–[19]. Bock *et al.* [16] made use of k-mers of AAs to infer PPIs by AA properties and Shen *et al.* [18] by k-mer frequencies. The former method made use of a small selected set of AA properties and an undefined method for reducing the feature space of the PPI representation to avoid the problem of having vectors of different length, due to different protein sequence lengths. The latter method only focuses on frequencies of AA triads that have been classified into groups of AAs with similar properties, and a newly proposed kernel method to circumvent the problem of symmetry of feature vectors (Protein1-Protein2 equals Protein2-Protein1). Pitre *et al.* [17] utilised the PAM120 similarity matrix to compare and score short AA sequences of individual partners of a hypothetical interaction with the sequences of proteins that are known to interact. Guo *et al.* [19] used a fixed set of seven distinct physicochemical properties to construct feature vectors based on auto covariance and thus circumvent the problem of vectors that differ in length. On the other hand, they did not address the problem of symmetry in protein pairs. Van Dijk *et al.* [20] focused on specific TF families and utilised short motif sequences found in sequences of TFs to predict specific TF interactions with the help of a random forest feature selection approach.

Our proposed method utilises only primary protein sequences to build a representation for a TF interaction pair without any additional prior knowledge to minimise the complexity in feature vector generation. The technique applied here for representing features is based on an averaging scheme of AA properties. Because the methodology is simple, it might obscure certain domain specific properties. This is particularly so as few residues are involved in the actual interactions, though the surrounding parts could play a role in the recognition of binding sites. Thus, most parts of the protein sequence are not necessary for the interaction and thus their influence to the averaged values might hamper the performance.

For a given AA sequence and an AA property *X*, we calculated the corresponding model feature as an average of *X* for individual AAs that the sequence comprises of. When we calculated such model feature, for example for the N-terminal and C-terminal parts of the sequence, we regarded these two averages as two distinct model feature values of the TF. Hence, even though these may appear to be the same features, in our model they are clearly distinct model features. Out of 3,186 such model features that we used for representing TF interacting pairs, our method selected a combination of 97 model features that resulted in the best prediction performance during 10-fold CV (see Figure 1). With the set of model features reduced to 97, our QDA-based method was able to achieve ∼85% accuracy in separating true from negative TF interactions based on a completely independent set of TF interactions.

In comparison, the performance of other approaches described above that are based on primary sequence alone to predict either PPI or TF interactions, achieved a prediction accuracy of around 80% (see Table S3). Several observations could be made on this point.

Bock *et al.* [16] obtained ∼80% accuracy for predicting PPIs from DIP with no preference on the organism. The negative examples were created by randomly shuffling AA sequences from DIP, while preserving AA composition and di– and tri-peptide ‘k-let’ frequencies. The method for creating negative examples only shuffled sequences from interacting proteins sampled from DIP, which hampers the variety of negative examples.

Shen *et al.* [18] achieved 83.9% accuracy for the prediction of human PPI. Here the training set was larger than the training set used in our method due to the utilised PPIs from HPRD. On the other hand, we used only TF interaction as opposed to all PPI. Negative examples were chosen randomly.

The yeast PPI prediction method by Guo *et al.* [19] achieved ∼87% in terms of accuracy. Their training set of PPI was again much larger than in our study. In addition, the ∼87% accuracy was only achieved with the negative examples chosen from non-co-localised proteins, meaning proteins that are not functional in the same cell compartment. On all other tested data sets they used the prediction accuracy drops below 80%. They tested their prediction method on yet another independent dataset and claimed ∼87% accuracy although the reported result was only a measure of the sensitivity, (only positive examples were input to the model for classification). Thus, the accuracy of their model could not be properly accessed.

Pietre *et al.* [17] achieved a prediction accuracy of 75% on PPI data for *Saccharomyces cerevisiae* gathered from DIP and MIPS. Their method does not require a set of negative PPI examples. It was evaluated on a set of 100 random positive examples from the databases and a negative set of 100 examples gathered from the literature.

Van Dijk *et al.* [20] predicted interaction between specific TF families and achieved for different families varying prediction accuracy ranging from 60–90%. A comparison of the performance of their approach to our method is difficult since it is not clear how they constructed the negative data set.

Analysis of published work in prediction of either PPIs or TF interactions from sequence data alone reveals, in general, four major drawbacks in methodology:

- Symmetry problem in the representation of pairs of interacting proteins
- Different feature-vector lengths, due to different protein sequence lengths
- Sequence similarity of proteins that bind identical protein partners, leading to biased models
- Missing negative set of protein interactions for training an appropriate model.

The first two problems deal with the representation of features, while the remaining two strongly affect the prediction performance of a system employed for classification. Our approach utilised a stringent methodology for the representation of a pair of TFs, thus not having a symmetrical effect while creating TF pairs (see Materials and Methods). Protein sequences of TFs vary in their length. In this study, a representation that is not dependent on the length of the AA sequence of a TF was implemented (see Materials and Methods). The nature of the feature representation approach utilised, ensures that a feature vector representation for any pair of TFs is always of the same size.

Proteins that have a high sequence similarity might have the same binding partners [52]. This could lead to a bias in performance assessment, which uses a CV scheme. For this reason we applied a filtering step before the model evaluation. We identified interactions where TFs with high sequences similarity (as defined previously in Materials and Methods) have identical binding partners and in such a case excluded all but one of the interactions (see Materials and Methods). In this manner we made sure that we did not introduce a bias based on sequence similarities of the TFs into our prediction methodology.

The problem that still exists is the relatively small number of training examples, both positive (interacting) and, in particular, negative (non-interacting) pairs. The lack of datasets of non-interacting TFs is a huge disadvantage. The same obstacles as in the PPI prediction task are evident [53]. Tuning parameters of machine learning algorithms on its own is not sufficient to compensate for inadequate real negative examples, which are necessary to develop high-performance classification systems. One common practice is to choose random negatives, assuming that in such random selection the proportion of actually interacting pairs is very small [18], [19], [54], [55]. Another common approach is to choose negative interaction partners that are not functional in the same cellular compartment [53], [56]. The latter approach can be argued to introduce a strong bias e.g. it leads to unrealistic high accuracy, and also cannot be applied in the case of predicting TF interactions, due to the localisation of TFs in the nucleus where they are functional. The random selection of negative examples in our study attempt to cover cases of non-interacting TF pairs with different levels of complexity (see Materials and Methods). The random selection of negatives TF pairs has its limitations. The performance of the system as presented here might not reflect the real performance, because some of the negatively denoted interactions can represent real interactions that are not yet experimentally verified or are not contained in the considered interaction databases. This has an influence on all performance measures, which might be in reality different. In particular, an in-depth experimental investigation into the group of false positive predictions might be of interest, because these would contain possible new true interactions that are not yet known. Nevertheless, the absence of a real set of non-interacting gene products, has been shown to be the bottleneck in all studies that dealt with either PPI or TF interaction prediction. In addition, during the task of predicting TF interactions, one has to deal with a relatively small set of positive interactions available for training as opposed to the larger number of positive examples in PPI prediction task. This is an additional shortcoming for developing models for predicting TF interactions with higher performance. It is also noteworthy what kinds of interactions are considered to form the positive set of interactions. Here, we are only interested in interaction types that can possibly lead to the formation of a protein (TF) complex. Thus, we only considered three types of interactions (MI:0195 - covalent binding, MI:0407 - direct interaction, MI:0915 - physical association) from the PSI-MI classification [46]. All interactions that we downloaded from the respective databases (see Materials and Methods) were filtered according to these three types of interactions. In this manner we excluded many interactions (e.g. chemical reactions between two proteins) that do not contribute to the formation of a protein complex. Unfortunately this reduced the already small set of available interactions between TFs even further.

It is known that not all residues in a protein are equally important as some are important for function and binding while others can be exchanged without such a loss of function [57]. The parts of a protein that interact with another protein are normally very short (often between 3 and 8 residues) [58]. The present study focused on the complete AA sequence of the TF. Further studies could incorporate methods for predicting the importance of certain AA residues in the sequence [59], e.g. through conservation analysis for protein-protein interfaces [60], [61] or protein domains.

Nevertheless, even though the task of predicting TF interactions is in a way more difficult than the prediction of general PPIs for the simple reason that negative samples have to be created by the same functional types of proteins (TFs) that exert their TF function in the same cellular compartment, the method applied here is able to achieve very good performance. The advantage of the method lies in its simplicity of feature representation and the number of features used. These results were achieved even though the resulting model did not require a large amount of prior knowledge such as sequence motifs, domains, and gene expression data, to be taken into account.

## Supporting Information

Table S1 **531 Features used for creating the feature vectors.** The table shows the 531 features that we used to create the feature vectors representing individual TFs. The individual columns hold the following information: feature number, the identifier of the AAIndex database, authors of Reference, title of reference, journal of reference.
(XLS)

Table S2 **97 features selected through feature selection.** The table shows the 97 features selected through the feature selection. Column 1 contains the feature number as selected by the algorithm for feature selection. Column 2 contains the corresponding feature position in the complete feature vector of 3,186 features. Column 3 shows if the feature is representative for TF 1 or 2, which form the interaction. Column 4 shows from which respective section of the sequence the feature was extracted. Column 5 shoes the respective feature number from Table S1.
(XLS)

Table S3 **Comparison of different classification methods.** The table shows the different methods used for classification of either PPI or TFs in comparison to our method. It provides, a short description of each method as well as the performance.
(XLS)
